# Hospital utilization rates following antipsychotic dose reductions: implications for tardive dyskinesia

**DOI:** 10.1186/s12888-018-1889-2

**Published:** 2018-09-24

**Authors:** Stanley N. Caroff, Fan Mu, Rajeev Ayyagari, Traci Schilling, Victor Abler, Benjamin Carroll

**Affiliations:** 1 Department of Psychiatry, Corporal Michael J. Crescenz VA Medical Center and the Perelman School of Medicine at the University of Pennsylvania, 3900 Woodland Avenue, Philadelphia, PA 19104 USA; 2 Analysis Group, 111 Huntington Ave, Boston, MA 02199 USA; 3 Teva Pharmaceutical Industries, 41 Moores Rd, Frazer, Malvern, PA 19355 USA

**Keywords:** Tardive dyskinesia; antipsychotic medication, Schizophrenia, Relapse, Healthcare burden

## Abstract

**Background:**

Data are limited on the benefits and risks of dose reduction in managing side effects associated with antipsychotic treatment. As an example, antipsychotic dose reduction has been recommended in the management of tardive dyskinesia (TD), yet the benefits of lowering doses are not well studied. However, stable maintenance treatment is essential to prevent deterioration and relapse in schizophrenia.

**Methods:**

A retrospective cohort study was conducted to analyze the healthcare burden of antipsychotic dose reduction in patients with schizophrenia. Medical claims from six US states spanning a six-year period were analyzed for ≥10% or ≥ 30% antipsychotic dose reductions compared with those from patients receiving a stable dose. Outcomes measured were inpatient admissions and emergency room (ER) visits for schizophrenia, all psychiatric disorders, and all causes, and TD claims.

**Results:**

A total of 19,556 patients were identified with ≥10% dose reduction and 15,239 patients with ≥30% dose reduction. Following a ≥ 10% dose reduction, the risk of an all-cause inpatient admission increased (hazard ratio [HR] 1.17; 95% confidence interval [CI] 1.11, 1.23; *P* < 0.001), and the risk of an all-cause ER visit increased (HR 1.09; 95% CI 1.05, 1.14; *P* < 0.001) compared with controls. Patients with a ≥ 10% dose reduction had an increased risk of admission or ER visit for schizophrenia (HR 1.27; 95% CI 1.19, 1.36; *P* < 0.001) and for all psychiatric disorders (HR 1.16; 95% CI 1.10, 1.23; *P* < 0.001) compared with controls. A dose reduction of ≥30% also led to an increased risk of admission for all causes (HR 1.23; 95% CI 1.17, 1.31; *P* < 0.001), and for admission or ER visit for schizophrenia (HR 1.31; 95% CI 1.21, 1.41; *P* < 0.001) or for all psychiatric disorders (HR 1.21; 95% CI 1.14, 1.29; *P* < 0.001) compared with controls. Dose reductions had no significant effect on claims for TD.

**Conclusion:**

Patients with antipsychotic dose reductions showed significant increases in both all-cause and mental health–related hospitalizations, suggesting that antipsychotic dose reductions may lead to increased overall healthcare burden in some schizophrenia patients. This highlights the need for alternative strategies for the management of side effects, including TD, in schizophrenia patients that allow for maintaining effective antipsychotic treatment.

**Electronic supplementary material:**

The online version of this article (10.1186/s12888-018-1889-2) contains supplementary material, which is available to authorized users.

## Background

Antipsychotic medication is the mainstay of treatment for schizophrenia [1]. Agreement on the therapeutic doses of antipsychotics has been controversial; doses are determined empirically based on assessment of efficacy and tolerability for individual patients [1]. When side effects emerge, management options include discontinuation of the current antipsychotic, switching to a different drug, or lowering the dose [2–4].

For example, tardive dyskinesia (TD), which occurs in up to 30% of patients receiving antipsychotics, is a serious side effect of antipsychotics for which dose reduction has been proposed [2–4]. However, controlled evidence on the effect of dose reduction for the management of TD remains limited. [5]. Although novel drugs for treatment of TD are now available, management begins with consideration of antipsychotic prescribing decisions. In non-psychotic patients with TD, antipsychotic drug discontinuation may be considered and drugs may be tapered off if clinically appropriate [6]. Early diagnosis and drug cessation may facilitate remission, but evidence is limited on whether or how often TD resolves, and how long it takes to do so, after discontinuation of antipsychotics [7, 8]. In psychotic patients, maintenance antipsychotic treatment is essential to prevent deterioration and relapse [9]. Approximately 50% of patients taking antipsychotics are being treated for schizophrenia [10], which has a relapse rate of 53% within 9 months after antipsychotic discontinuation [11]. Antipsychotic discontinuation, which is associated with increased risk of violence, incarceration, hospitalization, increased healthcare resource utilization, and interruption of rehabilitation efforts, may be impractical in most patients with schizophrenia [12, 13].

Although some published evidence supports increasing doses or switching to other antipsychotics to suppress TD symptoms [14–16], these changes may raise concerns about increasing acute extrapyramidal symptoms, limiting chances of TD reversibility, and possibly destabilizing psychiatric status. Dose reduction has also been recommended in past reviews of treatment options [5, 6, 17]. However, published meta-analyses have concluded that available data are insufficient to either support or refute treatment of TD by antipsychotic dose reduction [5, 18]. Furthermore, published studies of dose-reduction strategies to minimize the risk of developing extrapyramidal symptoms including TD, involving dose reductions of 50% or more, have been inconsistent but generally reported significant risks of psychotic exacerbation and relapse [9, 19–23].

To further address the risks of dose reduction as a recommended treatment intervention for side effects of antipsychotic treatment, including TD, we conducted a retrospective cohort study to analyze the healthcare burden in terms of utilization of hospital-based resources resulting from ≥10% and ≥ 30% reductions in antipsychotic doses for patients with schizophrenia.

## Methods

### Study objective and data sources

A large retrospective cohort study using electronic medical records was conducted to compare the risk of all-cause and mental health–related inpatient admissions and emergency room (ER) visits for schizophrenia patients who were treated with a stable dose versus those who experienced a dose reduction of an oral antipsychotic monotherapy.

Medicaid data was extracted from six US states (Iowa, Kansas, Mississippi, Missouri, New Jersey, and Wisconsin). These states were chosen based on the availability of data for the analysis. Each state has its own policies regarding use and sharing of their data, and these six states had agreements in place allowing this analysis to be performed compiling and using Medicaid data from each state. All states for which data were available were included. Data representing the most recent six years from each state were analyzed, the dates of which varied by state. The overall study period ranged from 2008 to 2017 for all medical records. The Medicaid records contained complete medical claims, including diagnosis, procedures, paid amounts, pharmaceutical claims, enrollment history, and patient demographics.

### Patient selection

Patients for the analysis met the following inclusion criteria: 18 years or older as of the index date; at least one diagnosis of schizophrenia in the most recent 6 years of data for each state; at least two fills of an oral antipsychotic after the first schizophrenia diagnosis; at least one antipsychotic monotherapy period that was ≥90 days with a stable dose; and a baseline period of at least 6 months of continuous enrollment prior to the index date.

Exclusion criteria included: patients receiving long-acting injectable antipsychotics; patients receiving more than one antipsychotic concurrently during the study period; and patients from New Jersey who turned 65 after 2012, as these patients were eligible for both Medicare and Medicaid, potentially preventing complete capture of drug claim information via the Medicaid database.

*“*Cases” were defined as patients with a stable-dose monotherapy period of ≥90 days who then experienced a dose reduction, defined as a reduction ≥10% from the stable dose. The date of the initial dose reduction was defined as the index date for cases. Controls were defined as patients with a stable-dose monotherapy period that lasted for ≥91 days without a dose reduction greater than 10%. The first prescription fill after the first 90-day stable-dose period was defined as the index date for controls. If a patient had multiple potential index dates that met all of the inclusion criteria, the index date used for the purposes of the study was randomly selected from the eligible potential index dates. A randomly chosen potential index date was used rather than the first such index date to avoid biasing the analysis towards earlier stages of the disease.

Cases and controls were matched 1:1 based on age, gender, state, healthcare plan type (health maintenance organization [HMO] vs fee-for-service [FFS]), index treatment (first- vs second-generation antipsychotic), and index year. All patients were followed for 2 years after the index date or until they were removed from the analysis due to either the earliest event of dose escalation (disqualification from dose-reduction analysis) or the end of eligibility (defined as any change outside of the original inclusion criteria, including medication switch, additional medication added, dose escalation, or occurrence of an outcome event). Mean duration of the follow-up period was also reported. Additional subgroup analyses were performed on the subset of cases experiencing a dose reduction of ≥30% together with matched controls.

The following patient characteristics were assessed for cases and controls during the baseline period or on the index date: characteristics (age, gender, state, and healthcare plan type); index year; index treatment; psychiatric comorbidity profile; Charlson Comorbidity Index (CCI) score, including individual comorbidities; psychotherapy; psychiatric medication; and observed disease duration, defined as the first observed schizophrenia diagnosis date prior to the index date.

### Outcome measurements

Patients were assessed for all-cause inpatient admission and ER visits as well as healthcare utilization related to an inpatient admission or ER visit for either schizophrenia or another psychiatric diagnosis. Schizophrenia inpatient admissions or ER visits were identified using the International Classification of Diseases, 9th revision (ICD-9) code 295.xx and ICD-10 codes F20.x and F25.x. Psychiatric inpatient admissions or ER visits were identified by ICD-9 or ICD-10 codes for schizophrenia-spectrum and other psychotic disorders, substance-related and addictive disorders, depressive disorders, bipolar and related disorders, trauma- and stressor-induced disorders, anxiety disorders, sleep-wake disorders, and personality disorders (Additional file 1: Table S1). Patients were also assessed for TD diagnosis, corresponding to ICD-9 code 333.85 (subacute dyskinesia due to drugs) or ICD-10 code G24.01 (drug-induced subacute dyskinesia).

### Statistical analysis

Descriptive statistics (mean, standard deviation, percentage) were used to describe patient demographics between case and control cohorts. Patient characteristics between cohorts were evaluated using Wilcoxon signed-rank tests for continuous variables and McNemar’s tests for dichotomous variables. For comparison of study outcomes (all-cause inpatient admission or ER visit, schizophrenia-related inpatient admission or ER visit, psychiatric-related inpatient admission or ER visit), Kaplan–Meier analyses with log-rank test were used. Multivariable Cox proportional hazard models were used to compare outcomes between cases and controls, adjusting for 19 covariates, including additional patient characteristics such as age (continuous), disease duration, CCI score, psychiatric comorbidity profile, psychotherapy use, and psychiatric medication use.

## Results

### Baseline characteristics

**Fig. 1:**
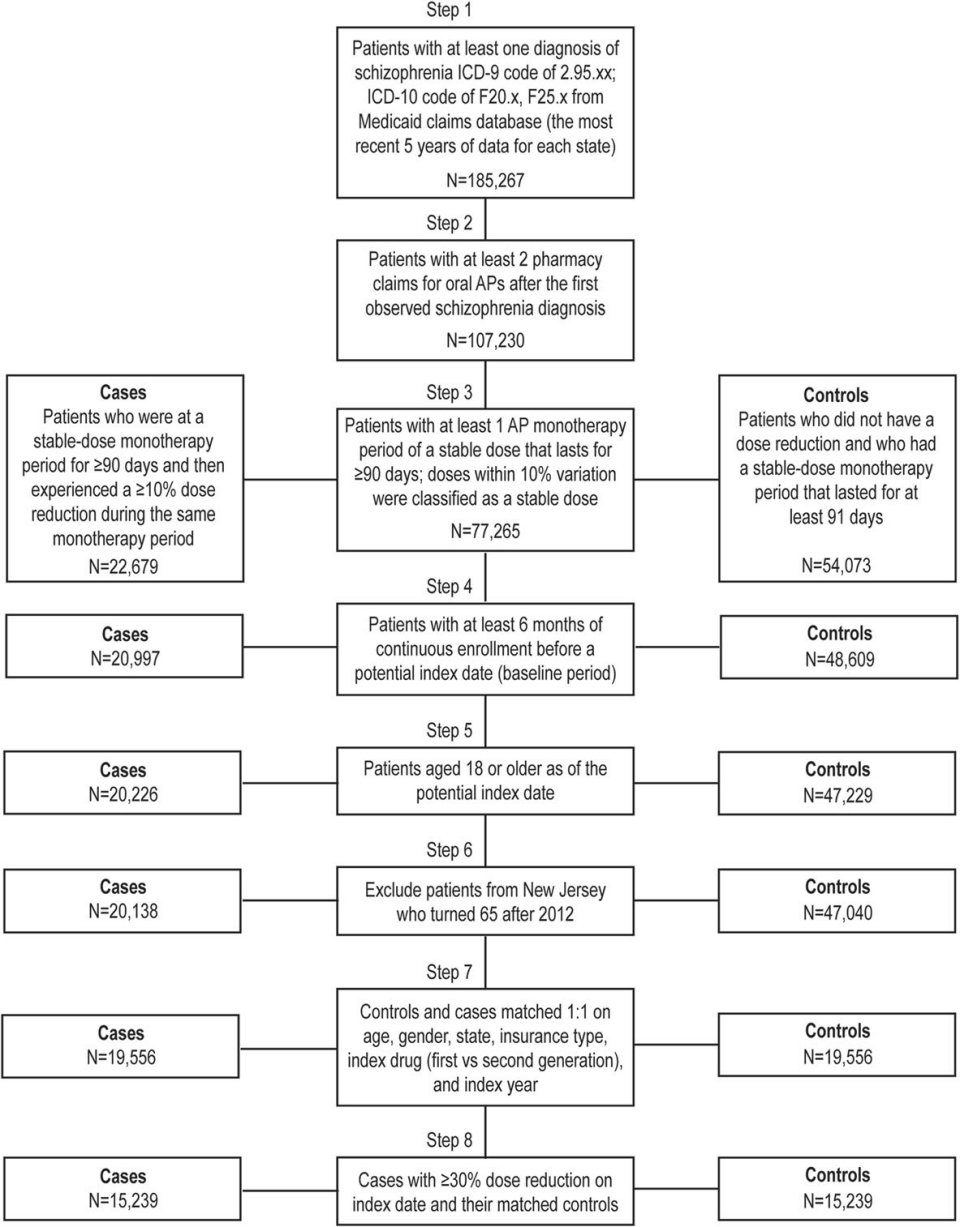
Patient Selection Flow Diagram. Patients were selected for case and control cohorts from a Medicaid claims database representing six US states and the most recent 6 years of data as detailed in Methods. AP, antipsychotic; ICD, International Classification of Diseases

A total of 185,267 patients were diagnosed with schizophrenia during the study period, and 19,556 case patients meeting the final inclusion criteria were matched 1:1 with control patients for the ≥10% dose-reduction analyses (Fig. 1). The resulting distribution of age, gender, state, and healthcare plan type were nearly identical between cases and controls. In both cohorts, the mean age was ~ 45.3 years and 52% were male, and ~ 44% and 18% of patients subscribed to FFS and HMO insurance plans (Table 1). Differences between the cohorts include the mean observed disease duration, which was 27 months for case patients and 20 months for control patients. In addition, the mean duration of follow-up time was 4.5 months for case patients and 8.0 months for control patients (Table 1). The demographics for the ≥30% dose-reduction cohorts are shown in Additional file 1: Table S2.

**Table 1:** Baseline demographics of ≥10% dose-reduction case and control cohorts

Characteristic	Case *N* = 19,556	Control *N* = 19,556	*P* Value
Demographics			
Age	45.3 ± 13.8	45.3 ± 13.8	0.86
Schizophrenia duration (months)	27.0 ± 17.9	20.1 ± 17.5	< 0.001
Male	10,075 (51.5%)	10,075 (51.5%)	–
Duration of follow-up (months)	4.5 ± 8.1	8.0 ± 11.7	< 0.001
Insurance type			
FFS	8562 (43.8%)	8562 (43.8%)	–
HMO	3603 (18.4%)	3603 (18.4%)	–
Mixed	7391 (37.8%)	7391 (37.8%)	–
State			
Iowa	1024 (5.2%)	1024 (5.2%)	–
Kansas	1294 (6.6%)	1294 (6.6%)	–
Mississippi	1761 (9.0%)	1761 (9.0%)	–
Missouri	7765 (39.7%)	7765 (39.7%)	–
New Jersey	5578 (28.5%)	5578 (28.5%)	–
Wisconsin	2134 (10.9%)	2134 (10.9%)	–
Index characteristics			
Index drug class			
First generation	2384 (12.2%)	2384 (12.2%)	–
Second generation	17,172 (87.8%)	17,172 (87.8%)	–
Index year			
2008	480 (2.5%)	480 (2.5%)	–
2009	1388 (7.1%)	1388 (7.1%)	–
2010	1799 (9.2%)	1799 (9.2%)	–
2011	2516 (12.9%)	2516 (12.9%)	–
2012	3280 (16.8%)	3280 (16.8%)	–
2013	3378 (17.3%)	3378 (17.3%)	–
2014	2196 (11.2%)	2196 (11.2%)	–
2015	2223 (11.4%)	2223 (11.4%)	–
2016	1910 (9.8%)	1910 (9.8%)	–
2017	376 (1.9%)	376 (1.9%)	
Comorbidity profile			
Substance-related and addictive disorders	4638 (23.7%)	4879 (25.0%)	< 0.01
Anxiety disorders	2973 (15.2%)	3264 (16.7%)	< 0.001
Bipolar and related disorders	4452 (22.8%)	4672 (23.9%)	< 0.01
Depressive disorders	4942 (25.3%)	5472 (28.0%)	< 0.001
Personality disorders	809 (4.1%)	757 (3.9%)	0.19
Schizophrenia-spectrum disorders (excluding schizophrenia)	2426 (12.4%)	2699 (13.8%)	< 0.001
Sleep–wake disorders	1516 (7.8%)	1565 (8.0%)	0.36
Tardive dyskinesia	37 (0.2%)	51 (0.3%)	0.17
Trauma- and stressor-related disorders	1312 (6.7%)	1446 (7.4%)	< 0.01
CCI	0.6 ± 1.2	0.6 ± 1.2	0.29
AIDS/HIV	238 (1.2%)	288 (1.5%)	< 0.05
Cancer	391 (2.0%)	399 (2.0%)	0.80
Cerebrovascular disease	765 (3.9%)	717 (3.7%)	0.21
Congestive heart failure	759 (3.9%)	722 (3.7%)	0.33
Chronic pulmonary disease	3901 (20.0%)	3836 (19.6%)	0.41
Dementia	512 (2.6%)	430 (2.2%)	< 0.01
Diabetes with chronic complication	791 (4.0%)	751 (3.8%)	0.30
Diabetes without chronic complication	3201 (16.4%)	3066 (15.7%)	0.06
Hemiplegia or paraplegia	220 (1.1%)	188 (1.0%)	0.12
Mild liver disease	800 (4.1%)	834 (4.3%)	0.40
Metastatic solid tumor	60 (0.3%)	50 (0.3%)	0.39
Myocardial infarction	189 (1.0%)	197 (1.0%)	0.72
Moderate or severe liver disease	68 (0.4%)	59 (0.3%)	0.48
Peptic ulcer disease	112 (0.6%)	111 (0.6%)	1.00
Peripheral vascular disease	883 (4.5%)	784 (4.0%)	< 0.05
Renal disease	559 (2.9%)	544 (2.8%)	0.67
Rheumatic disease	198 (1.0%)	221 (1.1%)	0.27
Psychotherapy			
Psychotherapy in crisis	36 (0.2%)	41 (0.2%)	0.65
Psychotherapy non-crisis	2587 (13.2%)	2791 (14.3%)	< 0.01
Psychoanalysis	0 (0%)	2 (0.01%)	0.48
Additional psychiatric medications			
ADHD medication	523 (2.7%)	513 (2.6%)	0.77
Anticholinergic	3998 (20.4%)	3465 (17.7%)	< 0.001
Antidepressant	9176 (46.9%)	9214 (47.1%)	0.63
Anxiety medication	4961 (25.4%)	4964 (25.4%)	0.98
Mood stabilizer	5980 (30.6%)	5388 (27.6%)	< 0.001
Sedative	1946 (10.0%)	2112 (10.8%)	< 0.01

Error represents standard deviation

*ADHD* attention-deficit/hyperactivity disorder; *AIDS* acquired immunodeficiency syndrome; *CCI* Charlson Comorbidity Index; *FFS* fee-for-service; *HIV* human immunodeficiency virus; *HMO* health maintenance organization

The distribution of index drug class was also identical between the case and control cohorts (Table 1). Eighty-eight percent of patients were using second-generation antipsychotics, with ~ 12% using first-generation antipsychotics. Cases had lower rates of psychiatric comorbidities and substance use disorders than controls, with the exception of personality disorders. In addition, the CCI score was also similar between cases and controls except for AIDS/HIV, dementia, and peripheral vascular disease**.** The number of TD claims reported for patients in both cohorts was extremely low, representing approximately 0.2–0.3% of the patient population.

### Dosing patterns

Table 2 shows the mean dose distribution between the case and control cohorts for the ten most commonly prescribed antipsychotic medications. Mean doses were higher in the case cohort versus the control cohort for all of the top ten medications.

**Table 2:** Dose distribution for ten most frequently used antipsychotics during stable-dose period among case and control cohorts

Drug	Case (N, %)	Case Stable Dose (mg), Mean ± SD	Control (N, %)	Control Stable Dose (mg), Mean ± SD
Risperidone	4564 (23.3)	7 ± 7	4135 (21.1)	5 ± 6
Quetiapine	3871 (19.8)	463 ± 278	3226 (16.5)	331 ± 254
Olanzapine	2558 (13.1)	22 ± 13	2422 (12.4)	17 ± 12
Aripiprazole	1838 (9.4)	29 ± 47	2712 (13.9)	22 ± 38
Paliperidone	1180 (6.0)	70 ± 68	1704 (8.7)	56 ± 63
Haloperidol	1472 (7.5)	31 ± 30	1396 (7.1)	22 ± 19
Ziprasidone	1185 (6.1)	147 ± 61	1026 (5.2)	125 ± 55
Clozapine	1251 (6.4)	407 ± 180	683 (3.5)	405 ± 190
Lurasidone	467 (2.4)	93 ± 42	820 (4.2)	68 ± 35
Fluphenazine	301 (1.5)	20 ± 13	277 (1.4)	15 ± 11

*SD* standard deviation

The mean and distribution of the percent reduction among cases for each of the top ten antipsychotics are shown in Table 3. Patients on all ten antipsychotic medications had similar dose reductions throughout; mean dose reductions ranged from 45.3% (ziprasidone) to 57.3% (paliperidone) (Table 3). For most antipsychotics, 25% of patients had a dose reduction of ~ 33% or less, and approximately half of patients had a dose reduction of 50% or less (Table 3).

**Table 3:** Dose reductions for patient percentiles for the ten most frequently used antipsychotic drugs

Drug	N	Mean	10th Percentile	25th Percentile	50th Percentile	75th Percentile	90th Percentile
Risperidone	4564	48.6%	25.0%	33.3%	50.0%	66.7%	83.3%
Quetiapine	3871	48.3%	25.0%	33.3%	50.0%	66.7%	80.0%
Olanzapine	2558	46.0%	25.0%	30.0%	50.0%	60.0%	75.0%
Aripiprazole	1838	47.4%	25.0%	33.3%	50.0%	60.0%	75.0%
Paliperidone	1180	57.3%	25.0%	33.3%	50.0%	90.0%	96.3%
Haloperidol	1472	54.9%	25.0%	34.3%	50.0%	75.0%	90.0%
Ziprasidone	1185	45.3%	25.0%	33.3%	50.0%	52.0%	75.0%
Clozapine	1251	47.3%	16.7%	23.1%	40.0%	75.0%	88.2%
Lurasidone	467	46.9%	25.0%	33.3%	50.0%	50.0%	75.0%
Fluphenazine	301	45.7%	25.0%	33.3%	50.0%	50.0%	70.0%

### All-cause hospital utilization

**Fig. 2:**
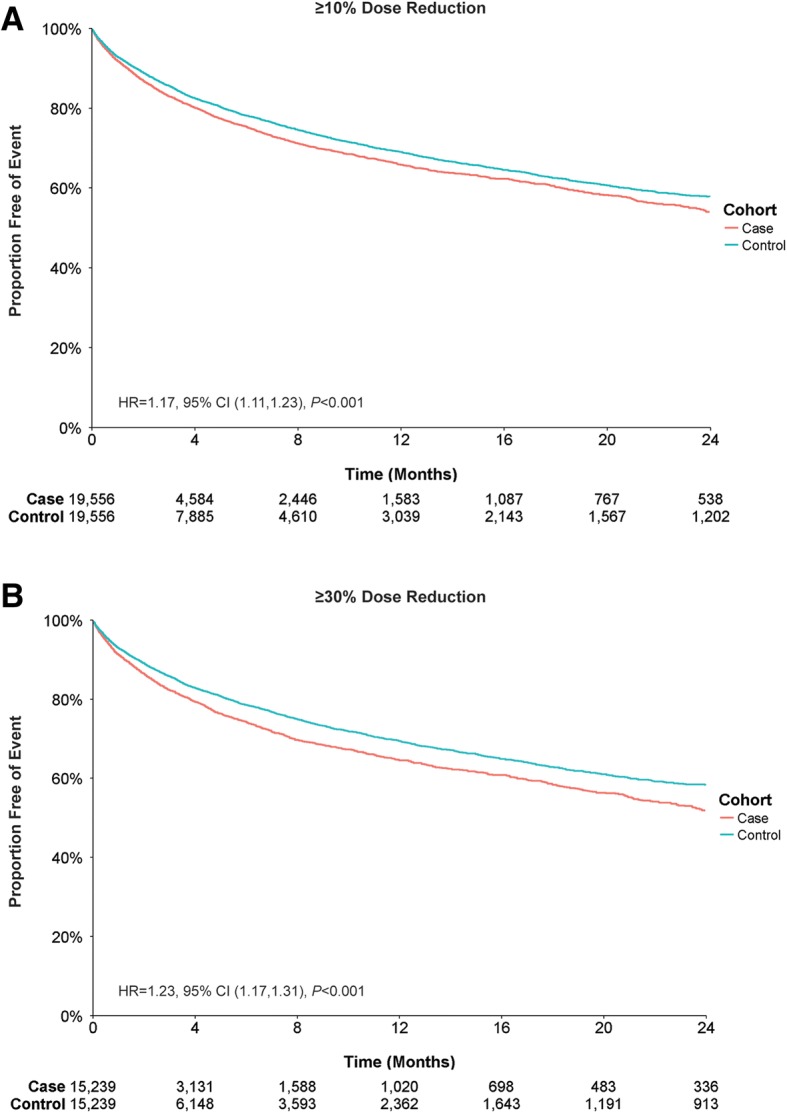
All-Cause Inpatient Admissions by Antipsychotic Dose Reduction. Patient claims were analyzed for all-cause inpatient admission for (**a**) ≥ 10% dose reductions or (**b**) ≥ 30% dose reductions of antipsychotic medication. Outcomes for case and control cohorts were assessed using Kaplan–Meier analysis and compared using a log-rank test. The number of patients at risk is represented for each time point. Case and control cohorts for ≥10%, *N* = 19,556 each; case and control cohorts for ≥30%, *N* = 15,239 each

Patients in the case and control cohorts were evaluated for all-cause healthcare utilization by assessing the likelihood of experiencing either an inpatient admission or an ER visit. For all-cause inpatient admissions, cases with a ≥ 10% dose reduction had a first-year event rate of 34.2%, compared with a 30.9% event rate for the control group, an absolute difference of 3.3% (Fig. 2a). Dose-reduction cases were more likely to have an inpatient admission compared with controls, and this increased likelihood persisted after adjusting for differences in baseline characteristics (Table 4).

**Table 4:** Multivariate cox regression analysis for comparison of outcome measures between case and control cohorts

Outcome	Dose Reduction	HR	95% CI	*P* Value
Inpatient admission	≥10%	1.17	1.11, 1.23	< 0.001
Inpatient admission	≥30%	1.23	1.17, 1.31	< 0.001
ER visit	≥10%	1.09	1.05, 1.14	< 0.001
ER visit	≥30%	1.14	1.08, 1.19	< 0.001
Schizophrenia admission or ER visit*	≥10%	1.27	1.19, 1.36	< 0.001
Schizophrenia admission or ER visit*	≥30%	1.31	1.21, 1.41	< 0.001
Psychiatric admission or ER visit*	≥10%	1.16	1.10, 1.23	< 0.001
Psychiatric admission or ER visit*	≥30%	1.21	1.14, 1.29	< 0.001
TD claim	≥10%	1.39	0.80, 2.41	0.24
TD claim	≥30%	1.60	0.86, 3.00	0.14

*See Additional file 1: Table S1 for ICD-9 and ICD-10 diagnostic codes

*CI* confidence interval; *ER* emergency room; *HR* hazard ratio; *ICD* International Classification of Diseases

Cases with a ≥ 30% dose reduction showed a slightly higher rate of all-cause inpatient admission compared with the ≥10% dose-reduction cases. The difference in first-year event rate between cases and controls was 4.9% (35.4% cases vs 30.5% controls) (Fig. 2b). Dose-reduction cases again showed an increased likelihood for inpatient admission compared with controls, which remained increased after adjusting for additional patient characteristics (Table 4).

**Fig. 3:**
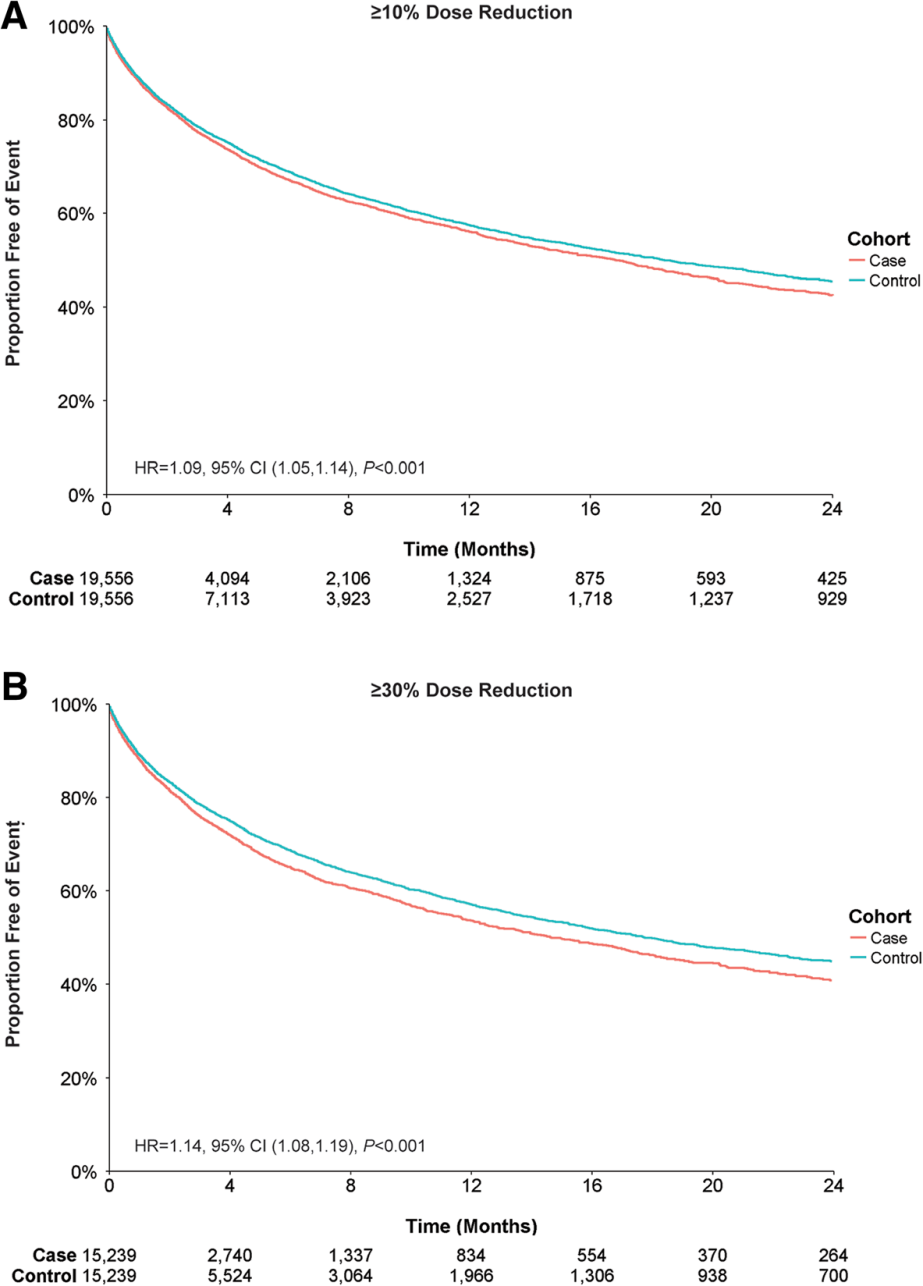
All-Cause Emergency Room Visits by Antipsychotic Dose Reduction. Patient claims were analyzed for all-cause emergency room visits for (**a**) ≥ 10% dose reductions or (**b**) ≥ 30% dose reductions of antipsychotic medication. Outcomes for case and control cohorts were assessed using Kaplan–Meier analysis and compared using a log-rank test. The number of patients at risk is represented for each time point. Case and control cohorts for ≥10%, *N* = 19,556 each; case and control cohorts for ≥30%, *N* = 15,239 each

For ER visits, cases with a ≥ 10% dose reduction had a first-year event rate difference of 1.4% (43.9% cases vs 42.5% controls) (Fig. 3a), whereas cases with a ≥ 30% dose reduction had a first-year event rate of difference of 3.4% (46.3% cases vs 42.9% controls) (Fig. 3b). Cases with ≥10% and ≥ 30% dose reductions were more likely to have an all-cause ER visit than controls, and this increased likelihood remained after adjusting for baseline patient characteristics (Table 4).

### Mental health–related hospital utilization

**Fig. 4:**
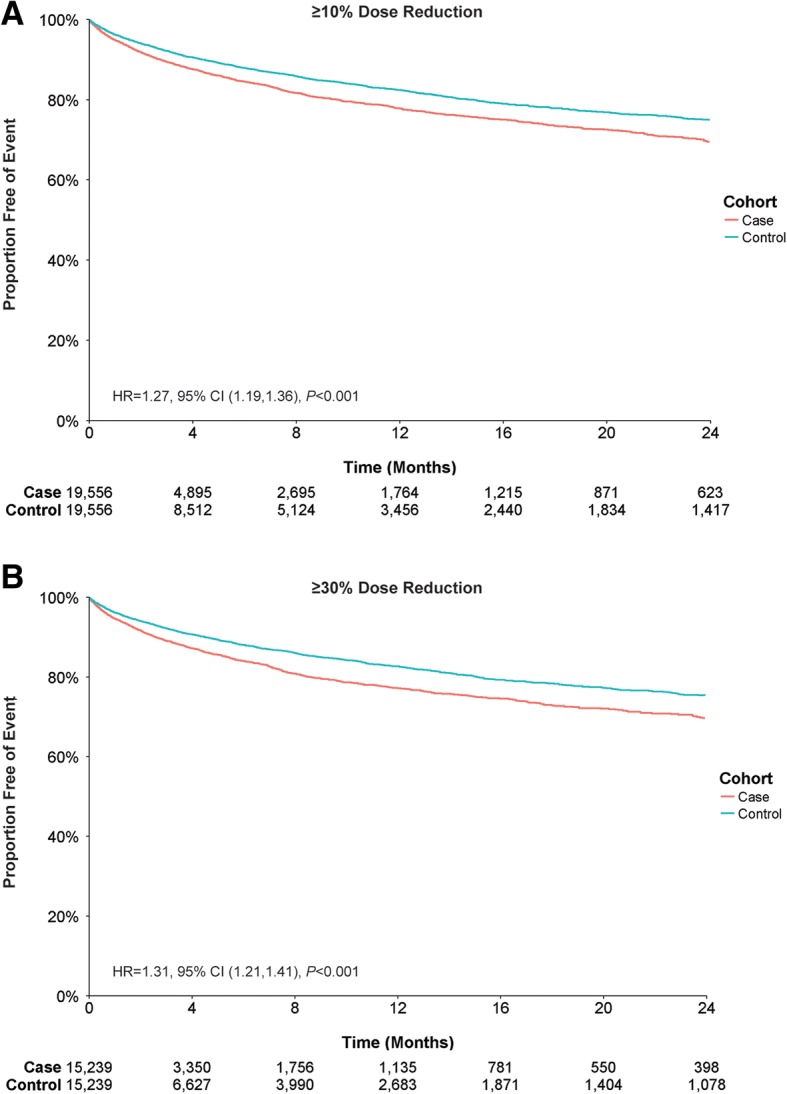
Inpatient Admissions or Emergency Room Visits for Schizophrenia by Antipsychotic Dose Reduction. Patient claims were analyzed for inpatient admissions or ER visits related to schizophrenia for (**a**) ≥ 10% dose reductions or (**b**) ≥ 30% dose reductions of antipsychotic medication. Outcomes for case and control cohorts were assessed using Kaplan–Meier analysis and compared using a log-rank test. The number of patients at risk is represented for each time point. Case and control cohorts for ≥10%, *N* = 19,556 each; case and control cohorts for ≥30%, *N* = 15,239 each

Patients in the case and control cohorts were also evaluated for mental health–related healthcare utilization by assessing the likelihood of experiencing an inpatient admission or ER visit for either a schizophrenia or psychiatric diagnosis (Additional file 1: Table S1). For schizophrenia, cases with a ≥ 10% dose reduction had a first-year event rate of 22.1%, compared with a 17.5% event rate for the control group, an absolute difference of 4.6% (Fig. 4a), and were more likely to have an inpatient admission or ER visit for schizophrenia than their control counterparts. After adjusting for differences in baseline characteristics, patients with a dose reduction of ≥10% were still more likely to have an inpatient admission or ER visit for schizophrenia than their counterparts with no dose reduction (Table 4)**.** Cases with a ≥ 30% dose reduction showed a slightly higher rate of schizophrenia admissions or ER visits than the ≥10% dose-reduction cases; the difference in first-year event rate between cases and controls was 5.5% (22.8% cases vs 17.3% controls) (Fig. 4b). These cases showed an increased likelihood for inpatient admission or ER visit for schizophrenia, which persisted after adjusting for additional patient characteristics (Table 4).

**Fig. 5:**
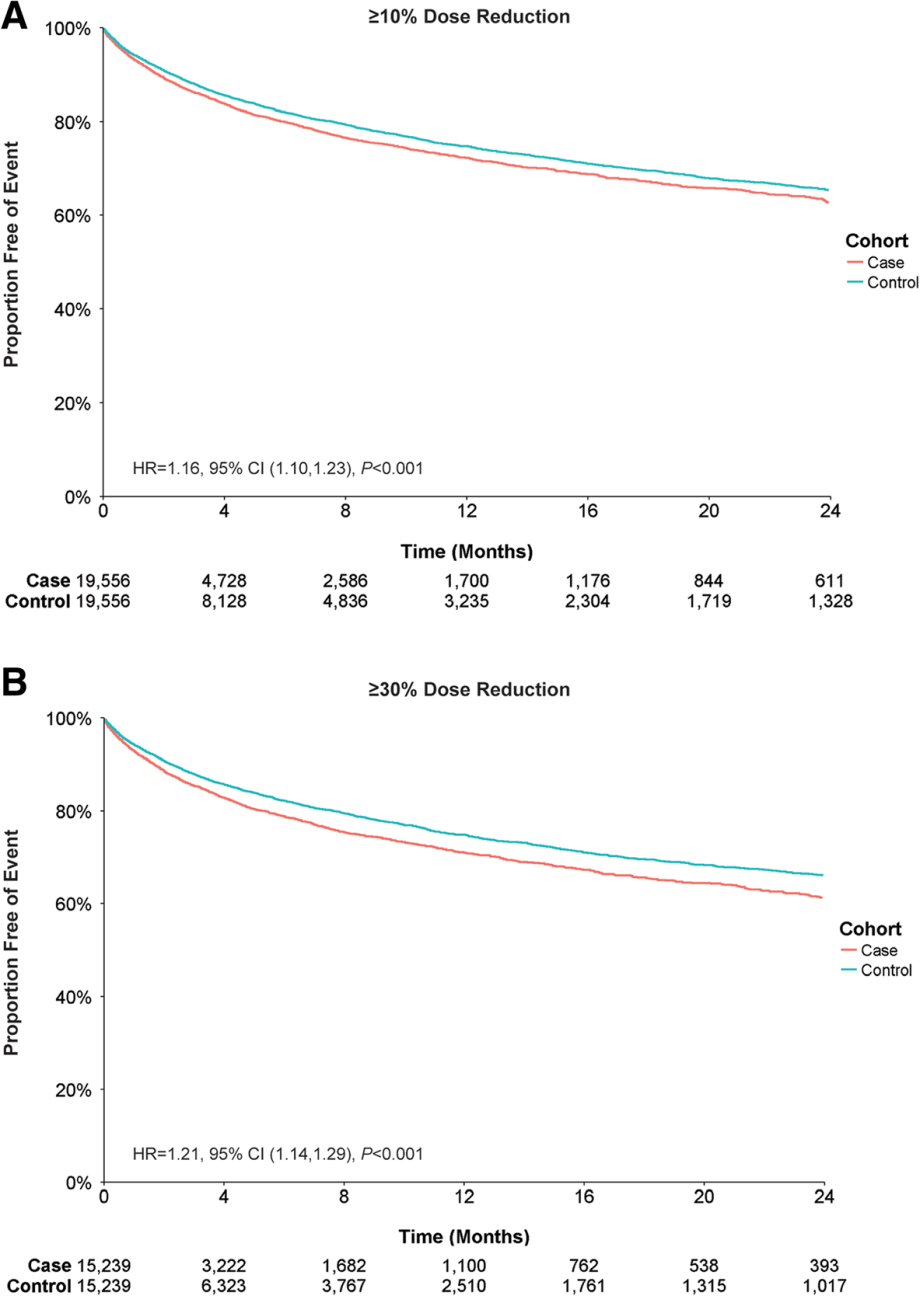
Inpatient Admissions or Emergency Room Visits for Psychiatric-Related Diagnosis by Antipsychotic Dose Reduction. Patient claims were analyzed for inpatient admissions or ER visits related to psychiatric diagnoses for (**a**) ≥ 10% dose reductions or (**b**) ≥ 30% dose reductions of antipsychotic medication. Outcomes for case and control cohorts were assessed using Kaplan–Meier analysis and compared using a log-rank test. The number of patients at risk is represented for each time point. Case and control cohorts for ≥10%, *N* = 19,556 each; case and control cohorts for ≥30%, *N* = 15,239 each

For psychiatric diagnoses, cases with a ≥ 10% or ≥ 30% dose reduction had a first-year event rate difference of 2.5% (27.7% cases vs 25.2% controls) and 3.8% (29.0% cases vs 25.2% controls) respectively (Fig. 5a, b). Cases with ≥10% and ≥ 30% dose reductions were more likely to have an inpatient admission or ER visit for psychiatric diagnoses than controls; when adjusted for baseline characteristics, this likelihood remained increased (Table 4).

### TD claims

Although analysis of TD claims was limited by the small sample size of TD claims, we assessed the existing claims for the emergence of any trends. Data from all patients in both cohorts, with and without TD at baseline, were analyzed with respect to the risk of a TD diagnosis during the study period. Cases with a ≥ 10% dose reduction did not show a lower risk of having a TD claim than controls (first-year event rate of 0.21% cases vs 0.24% controls; HR: 1.39, 95% CI 0.80, 2.41, *P =* 0.24). Patients with a ≥ 30% dose reduction also showed similar results (first-year event rate of 0.23% cases vs 0.22% controls; HR: 1.60, 95% CI 0.86, 3.00, *P =* 0.14). Both comparisons represented a non-significant difference in risk of having a TD claim after adjusting for baseline characteristics (Table 4).

Dose-reduction groups were also analyzed with respect to new cases of TD diagnosis during the study period compared with controls. To determine the risk of a new TD diagnosis, patients with TD at baseline were excluded (cases, *n* = 37; controls, *n* = 51). The resulting cases with a dose reduction did not have lower risk of having a TD claim compared with controls in the ≥10% group (first-year event rate of 0.16% cases vs 0.16% controls; HR: 1.66, 95% CI 0.87, 3.18, *P =* 0.12) and in the ≥30% group (first-year event rate of 0.18% cases vs 0.14% controls; HR: 1.84, 95% CI 0.89, 3.83, *P =* 0.10) after adjusting for baseline characteristics.

In addition, among patients with a TD claim during the baseline period, the percentages of patients having at least one additional TD claim during the first year of the study period were comparable between the dose-reduction cohort and the control cohort in both the ≥10% (21/37 [57%] vs 28/51 [55%], *P* = 0.33) and the ≥30% dose-reduction analyses (17/30 [57%] vs 21/40 [53%]; *P* = 0.46).

## Discussion

Patients with antipsychotic dose reductions showed significant increases in both all-cause and mental health–related admissions and ER visits. Absolute percentage differences in first-year hospital event rates were small (1–6%), but were statistically significant given the large sample size. The clinical meaningfulness of the observed differences should be considered by healthcare decision makers, because even a 1% difference in event rates reflected an additional 196 patients using hospital services during the study period. In addition, the shorter duration of follow-up observed for cases compared with controls suggests that dose reduction not only may increase the risk of hospital utilization, but also may result in shorter periods of treatment stability.

Hospital re-admissions for schizophrenia may be an indicator of an adverse outcome or failure of antipsychotic treatment after dose reductions. The chronic nature and severity of schizophrenia contribute to its large overall healthcare burden; direct healthcare costs for schizophrenia were estimated at 38 billion US dollars in 2013 [24]. Inpatient admissions represent one of the largest portions of the healthcare burden for schizophrenia, estimated in one study as 60–70% of direct costs [25]. The cost for schizophrenia patients who relapse is estimated to be two to five times higher than that for non-relapsed patients [26].

However, antipsychotic dose reduction in patients with schizophrenia may be necessary and appropriate for a variety of reasons; for example, patients in the dose-reduction cohort had higher mean doses to start with, as well as more cases of diabetes, which may have prompted reductions of antipsychotic doses. Although dose reduction has also been recommended to manage TD in previous practice guidelines for patients with schizophrenia who require maintenance antipsychotic treatment [5, 6], our results suggest that even relatively modest reductions in antipsychotic dosing, regardless of the rationale, may have significant adverse effects on outcomes for some patients. Therefore, prescribing decisions on maintenance antipsychotic treatment for preventing or treating TD should be carefully considered on an individualized basis for each patient. Apart from antipsychotic prescribing decisions, other measures, e.g., discontinuation or modification of anticholinergic drugs or treatment with vesicular monoamine transporter-2 inhibitors, may be considered in the management of TD.

The current study also attempted to evaluate antipsychotic dose reduction with respect to the diagnosis of TD and found no significant difference between stable dosing and dose reduction groups in either the number of new claims for TD during the study period based solely on the ICD-9 code of 333.85 and the ICD-10 code of G24.01 or in the persistence of a TD diagnosis following dose reduction. However, the duration of follow-up was significantly shorter in the dose-reduction group, and there were too few claims for TD in the databases compared with an expected prevalence of up to 30% in some previous studies [27]. This severely limits any interpretation of the relationship between dose reduction and the treatment of TD that was present at baseline, as well as the risk of new cases of TD developing in the total patient population. We suspect that TD is vastly underreported in claims databases [28], possibly due to complacency or a lack of clinical awareness, screening, approved treatments, standardization of the diagnosis or clarity regarding ICD codes. This study therefore highlights the need to document TD in a more systematic fashion. In addition, further study is needed to evaluate whether antipsychotic dose reduction is an effective treatment strategy for established cases of TD.

Strengths of the current study include the use of large patient cohorts with a 2-year follow-up period. In addition, claims data were analyzed from multiple states, with closely matched case and control groups. Study limitations include the possibility that the dose-reduction group had more hospitalizations because it represented a more-severe or chronically ill population compared with controls as suggested by the slightly longer duration of underlying schizophrenia among the case patients, the higher mean doses of antipsychotics they received, and the higher number of patients receiving clozapine at baseline. However, patients with more-severe disease would have been less likely to have doses reduced by prescribers and qualify as cases. In addition, more-severely ill patients were excluded from the analysis if they received multiple antipsychotics during the study period, had recent increases in doses (dose-reduction group), or were receiving long-acting injectable antipsychotics, all reflective of more-resistant disease. Comorbid psychiatric diagnoses that contribute to severity and hospitalizations, especially substance use, were actually more common among controls and may have biased results against our findings, resulting in worse outcomes among controls. Finally, outcome measures were controlled for numerous covariates in the Cox model that may reflect severity of illness, including psychiatric, substance use and medical co-morbidities, illness duration, and concomitant medication use. Nonadherence to treatment is another major contributor to relapse and hospitalization that may have affected differences in outcomes. However, enrollment criteria screened against including recognized nonadherent patients; patients were excluded if they received long-acting injectable antipsychotics or if they had fewer than 90 days of stable prescription monotherapy with antipsychotics prior to the index study date. Even if the dose reduction group showed greater rates of hospitalization because of nonadherence after the index date, that would still be clinically meaningful evidence suggesting untoward treatment effects of dose reduction. The high attrition during the 2 years of the study and the difference in attrition rates between cohorts were due to the cohort definitions, the differences in outcomes, and possibly nonadherence. Specifically, patients in the dose-reduction and control groups were censored at any change in antipsychotic use outside of the original inclusion criteria or after occurrence of an outcome event. The attrition and censoring of patients were accounted for in the Kaplan–Meier analyses and in the Cox models, which are statistical tools designed to handle censored or truncated time-to-event data [29]. Finally, given the retrospective nature of this claims-based study, we could not confirm the reasons for antipsychotic dose reductions and whether dose reduction affected outcomes positively in relation to other clinical domains.

## Conclusion

Results from this study show statistically significant differences in hospital utilization event rates, and suggest that dose reductions of antipsychotics may destabilize psychiatric status and lead to increased hospitalization rates in some patients with schizophrenia, resulting in damaging effects on recovery and adding to overall healthcare costs. Dose reductions had no effect on claims for TD, but this finding was confounded by the limited duration of follow-up and underreporting of the diagnosis. Therefore, decisions on dose reduction of antipsychotics in schizophrenia patients who require maintenance antipsychotic treatment, whether to prevent or reduce TD symptoms or for other reasons, should be carefully considered on an individualized basis. These results may highlight the need for alternative management strategies apart from dose reduction to reduce distressing side effects that would allow physicians to maintain effective maintenance treatment for patients with schizophrenia.

## Additional file

Additional file 1: **Table S1.** Diagnostic Codes for Schizophrenia and Psychiatric Relapse. Listing of ICD-9 and ICD-10 codes for disorders identified as causes for hospitalization and ER visits. **Table S2.** Baseline Demographics of ≥30% Dose-Reduction Case and Control Cohorts. Comparison of baseline demographic data between cases undergoing ≥30% dose reduction of antipsychotics versus controls on stable doses. (DOCX 25 kb)

## Abbreviation

ADHD: Attention-deficit/hyperactivity disorder
AIDS: Acquired immunodeficiency syndrome
CCI: Charlson Comorbidity Index
CI: Confidence interval
ER: Emergency room
FFS: Fee-for-service
HIV: Human immunodeficiency virus
HMO: Health maintenance organization
HR: Hazard ratio
ICD: International Classification of Diseases
TD: Tardive dyskinesia

## References

[1] Lehman AF, Lieberman JA, Dixon LB, McGlashan TH, Miller AL, Perkins DO (2004). Practice guidelines: practice guideline for the treatment of patients with schizophrenia (second edition). Am J Psychiatry.

[2] Aquino CC, Lang AE (2014). Tardive dyskinesia syndromes: current concepts. Parkinsonism Relat Disord.

[3] Caroff SN, Campbell EC (2016). Drug-induced extrapyramidal syndromes: Implications for contemporary practice. Psychiatr Clin North Am.

[4] Waln O, Jankovic J. An update on tardive dyskinesia: from phenomenology to treatment. Tremor Other Hyperkinet Mov (N Y). 2013:3. 10.7916/D88P5Z71 PMC3709416 23858394

[5] Bhidayasiri R, Fahn S, Weiner WJ, Gronseth GS, Sullivan KL, Zesiewicz TA (2013). Evidence-based guideline: treatment of tardive syndromes: report of the guideline development Subcommittee of the American Academy of neurology. Neurology.

[6] American Psychiatric Association. Tardive dyskinesia: a task force report of the American Psychiatric Association. American Psychiatric Association, Washington, DC, 1992.

[7] Glazer WM, Moore DC, Schooler NR, Brenner LM, Morgenstern H (1984). Tardive dyskinesia. A discontinuation study. Arch Gen Psychiatry.

[8] Zutshi D, Cloud LJ, Factor SA (2014). Tardive syndromes are rarely reversible after discontinuing dopamine receptor blocking agents: experience from a university-based movement disorder clinic. Tremor Other Hyperkinet Mov (N Y)..

[9] Schooler NR (1991). Maintenance medication for schizophrenia: strategies for dose reduction. Schizophr Bull.

[10] Dhir A, Schilling T, Abler V, Potluri R, Carroll B. estimation of epidemiology of tardive dyskinesia incidence and prevalence in the United States. The American Academy of Neurology annual meeting; April 22–28, 2017; Boston, MA.

[11] Gilbert PL, Harris M, McAdams L, Jeste DV (1995). Neuroleptic withdrawal in schizophrenic patients: a review of the literature. Arch Gen Psychiatry.

[12] Sariah AE, Outwater AH, Malima KI (2014). Risk and protective factors for relapse among individuals with schizophrenia: a qualitative study in Dar Es Salaam, Tanzania. BMC Psychiatry..

[13] Pigott TA, Carson WH, Saha AR, Torbeyns AF, Stock EG, Ingenito GG, Apriprazole Study Group (2003). Aripiprazole for the prevention of relapse in stabilized patients with chronic schizophrenia: a placebo-controlled 26-week study. J Clin Psychiatry.

[14] Brar JS, Parepally H, Chalasani L, Gopalani A, Appel N, Chengappa KN (2008). The impact of olanzapine on tardive dyskinetic symptoms in a state hospital population. Ann Clin Psychiatry.

[15] Caroff SN, Davis VG, Miller DD, Davis SM, Rosenheck RA, McEvoy JP (2011). Treatment outcomes of patients with tardive dyskinesia and chronic schizophrenia. J Clin Psychiatry..

[16] Mentzel Charlotte L., Bakker P. Roberto, van Os Jim, Drukker Marjan, Matroos Glenn E., Hoek Hans W., Tijssen Marina A. J., van Harten Peter N. (2017). Effect of Antipsychotic Type and Dose Changes on Tardive Dyskinesia and Parkinsonism Severity in Patients With a Serious Mental Illness. The Journal of Clinical Psychiatry.

[17] Kane JM, Woerner M, Sarantakos S, Kinon B, Lieberman J. Do low dose neuroleptics prevent or ameliorate tardive dyskinesia? In: Casey D, Gardos G, editors. Tardive Dyskinesia and Neuroleptics: From Dogma to Reason. Washington, D.C.: American Psychiatric Press, Inc. p. 100–7.

[18] Soares KV, McGrath JJ (1999). The treatment of tardive dyskinesia--a systematic review and meta-analysis. Schizophr Res.

[19] Tarsy D, Baldessarini RJ (2006). Epidemiology of tardive dyskinesia: is risk declining with modern antipsychotics?. Mov Disord.

[20] Davis JM, Matalon L, Watanabe MD, Blake L, Metalon L (1994). Depot antipsychotic drugs. Place in therapy Drugs.

[21] Wang CY, Xiang YT, Cai ZJ, Weng XY, Bo QJ, Zhao JP (2010). Risperidone maintenance treatment in schizophrenia: a randomized, controlled trial. Am J Psychiatry.

[22] Cookson IB (1987). The effects of a 50% reduction of cis(z)-flupenthixol decanoate in chronic schizophrenic patients maintained on a high dose regime. Int Clin Psychopharmacol.

[23] Kane JM, Rifkin A, Woerner M, Reardon G, Sarantakos S, Schiebel D, Ramos-Lorenzi J (1983). Low-dose neuroleptic treatment of outpatient schizophrenics. I. Preliminary results for relapse rates. Arch Gen Psychiatry.

[24] Cloutier M, Aigbogun MS, Guerin A (2016). The economic burden of schizophrenia in the United States in 2013. J Clin Psychiatry..

[25] Svarstad BL, Shireman TI, Sweeney JK (2001). Using drug claims data to assess the relationship of medication adherence with hospitalization and costs. Psychiatr Serv.

[26] Ascher-Svanum H, Zhu B, Faries DE, Salkever D, Slade EP, Peng X, Conley RR (2010). The cost of relapse and the predictors of relapse in the treatment of schizophrenia. BMC Psychiatry.

[27] Carbon M, Hsieh CH, Kane JM, Correll CU (2017). Tardive dyskinesia prevalence in the period of second-generation antipsychotic use: a meta-analysis. J Clin Psychiatry..

[28] Cortese L, Jog M, McAuley TJ, Kotteda V, Costa G (2004). Assessing and monitoring antipsychotic-induced movement disorders in hospitalized patients: a cautionary study. Can J Psychiatr.

[29] Kleinbaum D, Klein M (2012). Survival analysis: a self-learning text.

