# Supplementary material for: Hospital utilization rates following antipsychotic dose reductions: implications for tardive dyskinesia
Source: BMC Psychiatry. 2018 Sep 24;18:306. doi: 10.1186/s12888-018-1889-2 (PMC6154822; doi:10.1186/s12888-018-1889-2)
Supplement: Supplementary file 1 — Table S1. Diagnostic Codes for Schizophrenia and Psychiatric Relapse. Listing of ICD-9 and ICD-10 codes for disorders identified as causes for hospitalization and ER visits. Table S2. Baseline Demographics of ≥30% Dose-Reduction Case and Control Cohorts. Comparison of baseline demographic data between cases undergoing ≥30% dose reduction of antipsychotics versus controls on stable doses. (DOCX 25 kb) [file 12888_2018_1889_MOESM1_ESM.docx]

**Table S1. Diagnostic Codes for Schizophrenia and Psychiatric Relapse.**

| Condition | ICD-9 Code | ICD-10 Code |
| --- | --- | --- |
| **Schizophrenia relapse** |  |  |
| Schizophrenia | 295.xx | F20, F25 |
| **Psychiatric relapse** |  |  |
| Schizophrenia-spectrum and other psychotic disorders (excluding schizophrenia) | 293.81, 293.82, 293.89, 297.1, 298.8, 298.9, 301.22 | F062, F060, F061, F53, F22, F23, F29, F21 |
| Substance-related and addictive disorders | 291.81, 291.9, 292.0, 292.89, 292.9, 303.00, 303.90, 304.00, 304.10, 304.20, 304.30, 304.40, 304.50, 304.60, 304.90, 305.00, 305.1, 305.20, 305.30, 305.40, 305.50, 305.60, 305.70, 305.90 | F10, F11, F12, F13, F14, F15, F17, F18, F19 |
| Depressive disorders | 296.2, 296.3, 300.4, 625.4, 311 | F32, F33, F341, N943 |
| Bipolar and related disorders | 293.83, 296.4, 296.5, 296.7, 296.80, 296.89, 301.13 | F0630, F30, F31, F340 |
| Trauma- and stressor-related disorders | 308.3, 309.81, 309.0, 309.24, 309.28, 309.3, 309.4, 309.9, 309.89, 313.89 | F43, F938, F941, F988 |
| Anxiety disorders | 309.21, 312.23, 300.29, 300.23, 300.01, 300.22, 300.02, 293.84, 300.09, 300.00 | F930, F91, F40, F41, F064 |
| Sleep–wake disorders | 307.45, 307.46, 307.47, 327.21, 327.23, 327.24, 327.25, 327.26, 327.42, 333.94, 347.00, 347.01, 347.10, 780.52, 780.54, 780.57, 780.59, 786.04 | F51, F950, G47, G2581, R063 |
| Personality disorders | 301.0, 301.1, 301.20, 301.4, 301.5, 301.6, 301.81, 301.82, 301.83, 301.89, 301.9 | F60 |

ICD, International Classification of Diseases.

**Table S2. Baseline Demographics of ≥30% Dose-Reduction Case and Control Cohorts.**

| Characteristic | Case  N=15239 | Control  N=15239 | *P* Value |
| --- | --- | --- | --- |
| **Demographics** |  |  |  |
| Age | 45.2 ± 13.8 | 45.2 ± 13.8 | 0.47 |
| Schizophrenia duration (months) | 27.0 ± 17.9 | 20.2 ± 17.5 | <0.001 |
| Male | 7,772 (51.0%) | 7,772 (51.0%) | - |
| Duration of follow-up (months) | 4.0 ± 7.5 | 8.0 ± 11.6 | <0.001 |
| Insurance type |  |  |  |
| FFS | 6,565 (43.1%) | 6,565 (43.1%) | - |
| HMO | 2,808 (18.4%) | 2,808 (18.4%) | - |
| Mixed | 5,866 (38.5%) | 5,866 (38.5%) | - |
| State |  |  |  |
| Iowa | 805 (5.3%) | 805 (5.3%) | - |
| Kansas | 1,026 (6.7%) | 1,026 (6.7%) | - |
| Mississippi | 1,430 (9.4%) | 1,430 (9.4%) | - |
| Missouri | 6,048 (39.7%) | 6,048 (39.7%) | - |
| New Jersey | 4,314 (28.3%) | 4,314 (28.3%) | - |
| Wisconsin | 1,616 (10.6%) | 1,616 (10.6%) | - |
| **Index Characteristics** |  |  |  |
| Index drug class |  |  |  |
| First generation | 1,972 (12.9%) | 1,972 (12.9%) | - |
| Second generation | 13,267 (87.1%) | 13,267 (87.1%) | - |
| Index year |  |  |  |
| 2008 | 364 (2.4%) | 364 (2.4%) |  |
| 2009 | 1,037 (6.8%) | 1,037 (6.8%) | - |
| 2010 | 1,374 (9.0%) | 1,374 (9.0%) | - |
| 2011 | 1,955 (12.8%) | 1,955 (12.8%) | - |
| 2012 | 2,584 (17.0%) | 2,584 (17.0%) | - |
| 2013 | 2,621 (17.2%) | 2,621 (17.2%) | - |
| 2014 | 1,729 (11.4%) | 1,729 (11.4%) | - |
| 2015 | 1,739 (11.4%) | 1,739 (11.4%) | - |
| 2016 | 1,530 (10.0%) | 1,530 (10.0%) | - |
| 2017 | 306 (2.0%) | 306 (2.0%) |  |
| **Comorbidity Profile** |  |  |  |
| Substance-related and addictive disorders | 3,729 (24.5%) | 3,837 (25.2%) | 0.15 |
| Anxiety disorders | 2,407 (15.8%) | 2,575 (16.9%) | <0.01 |
| Bipolar and related disorders | 3,562 (23.4%) | 3,704 (24.3%) | 0.05 |
| Depressive disorders | 3,992 (26.2%) | 4,312 (28.3%) | <0.001 |
| Personality disorders | 628 (4.1%) | 606 (4.0%) | 0.54 |
| Schizophrenia-spectrum disorders (excluding schizophrenia) | 1,961 (12.9%) | 2,103 (13.8%) | <0.05 |
| Sleep–wake disorders | 1,208 (7.9%) | 1,242 (8.2%) | 0.48 |
| Tardive dyskinesia | 30 (0.2%) | 40 (0.3%) | 0.28 |
| Trauma- and stressor-related disorders | 1,063 (7.0%) | 1,158 (7.6%) | <0.05 |
| **CCI Score** | 0.65 ± 1.3 | 0.63 ± 1.3 | <0.05 |
| AIDS/HIV | 192 (1.3%) | 228 (1.5%) | 0.08 |
| Cancer | 303 (2.0%) | 309 (2.0%) | 0.84 |
| Cerebrovascular disease | 627 (4.1%) | 555 (3.6%) | <0.05 |
| Congestive heart failure | 633 (4.2%) | 576 (3.8%) | 0.09 |
| Chronic pulmonary disease | 3,086 (20.3%) | 3,012 (19.8%) | 0.29 |
| Dementia | 403 (2.6%) | 331 (2.2%) | <0.01 |
| Diabetes with chronic complication | 632 (4.2%) | 598 (3.9%) | 0.33 |
| Diabetes without chronic complication | 2,503 (16.4%) | 2,386 (15.7%) | 0.06 |
| Hemiplegia or paraplegia | 187 (1.2%) | 145 (1.0%) | <0.05 |
| Mild liver disease | 650 (4.3%) | 661 (4.3%) | 0.78 |
| Metastatic solid tumor | 52 (0.3%) | 43 (0.3%) | 0.41 |
| Myocardial infarction | 163 (1.1%) | 157 (1.0%) | 0.78 |
| Moderate or severe liver disease | 55 (0.4%) | 39 (0.3%) | 0.12 |
| Peptic ulcer disease | 91 (0.6%) | 89 (0.6%) | 0.94 |
| Peripheral vascular disease | 683 (4.5%) | 625 (4.1%) | 0.1 |
| Renal disease | 439 (2.9%) | 430 (2.8%) | 0.78 |
| Rheumatic disease | 170 (1.1%) | 162 (1.1%) | 0.69 |
| **Psychotherapy** |  |  |  |
| Psychotherapy in crisis | 29 (0.2%) | 32 (0.2%) | 0.8 |
| Psychotherapy non-crisis | 2,073 (13.6%) | 2,196 (14.4%) | <0.05 |
| Psychoanalysis | 0 (0%) | 2 (0.01%) | 0.48 |
| **Psychiatric Medication** |  |  |  |
| ADHD medication | 420 (2.8%) | 397 (2.6%) | 0.42 |
| Anticholinergic | 3,094 (20.3%) | 2,744 (18.0%) | <0.001 |
| Antidepressant | 7,211 (47.3%) | 7,217 (47.4%) | 0.94 |
| Anxiety medication | 3,945 (25.9%) | 3,876 (25.4%) | 0.33 |
| Mood stabilizer | 4,720 (31.0%) | 4,247 (27.9%) | <0.001 |
| Sedative | 1,568 (10.3%) | 1,674 (11.0%) | <0.05 |

Error represents standard deviation.

AIDS, acquired immunodeficiency syndrome; ADHD, attention-deficit/hyperactivity disorder; CCI, Charlson Comorbidity Index; FFS, fee for service; HIV, human immunodeficiency virus; HMO, health maintenance organization.
